# Effect of tetracycline hydrochloride application on dental pulp stem cell metabolism–booster or obstacle for tissue engineering?

**DOI:** 10.3389/fphar.2023.1277075

**Published:** 2023-09-28

**Authors:** Wang Wang, Jiangling Sun, Ghazal Aarabi, Ulrike Peters, Frank Fischer, Jan Klatt, Martin Gosau, Ralf Smeets, Thomas Beikler

**Affiliations:** ^1^ Department of Periodontics, Preventive and Restorative Dentistry, University Medical Center Hamburg-Eppendorf, Hamburg, Germany; ^2^ Department of Science and Education, Guiyang Stomatological Hospital, Guiyang, Guizhou, China; ^3^ Department of Oral and Maxillofacial Surgery, University Medical Center Hamburg-Eppendorf, Hamburg, Germany; ^4^ Department of Oral and Maxillofacial Surgery, Division of Regenerative Orofacial Medicine, University Medical Center Hamburg-Eppendorf, Hamburg, Germany

**Keywords:** human dental pulp stem cells, tetracycline hydrochloride, tissue engineering, scaffold, proliferation ability, cellular differentiation

## Abstract

**Introduction:** Stem cells and scaffolds are an important foundation and starting point for tissue engineering. Human dental pulp stem cells (DPSC) are mesenchymal stem cells with self-renewal and multi-directional differentiation potential, and are ideal candidates for tissue engineering due to their excellent biological properties and accessibility without causing major trauma at the donor site. Tetracycline hydrochloride (TCH), a broad-spectrum antibiotic, has been widely used in recent years for the synthesis of cellular scaffolds to reduce the incidence of postoperative infections.

**Methods:** In order to evaluate the effects of TCH on DPSC, the metabolism of DPSC in different concentrations of TCH environment was tested. Moreover, cell morphology, survival rates, proliferation rates, cell migration rates and differentiation abilities of DPSC at TCH concentrations of 0–500 μg/ml were measured. Phalloidin staining, live-dead staining, MTS assay, cell scratch assay and real-time PCR techniques were used to detect the changes in DPSC under varies TCH concentrations.

**Results:** At TCH concentrations higher than 250 μg/ml, DPSC cells were sequestered, the proportion of dead cells increased, and the cell proliferation capacity and cell migration capacity decreased. The osteogenic and adipogenic differentiation abilities of DPSC, however, were already inhibited at TCH con-centrations higher than 50 μg/ml. Here, the expression of the osteogenic genes, runt-related transcription factor 2 (RUNX2) and osteocalcin (OCN), the lipogenic genes lipase (LPL), as well as the peroxisome proliferator-activated receptor-γ (PPAR-γ) expression were found to be down-regulated.

**Discussion:** The results of the study indicated that TCH in concentrations above 50 µg/ml negatively affects the differentiation capability of DPSC. In addition, TCH at concentrations above 250 µg/ml adversely affects the growth status, percentage of living cells, proliferation and migration ability of cells.

## 1 Introduction

Tissue engineering is currently one of the fastest growing fields in medical and dental science. By applying principles of tissue engineering, trauma or genetic defects of tissue including skin, nerve, tendon or bone can be regenerated (Gomri et al., 2022). Tissue engineering generally consists of three basic components: suitable stem cells, scaffolds, and growth factors (La Noce et al., 2014; Anitua et al., 2018). Stem cells are a type of cell with a high proliferative capacity as well as differentiation capability and can be employed to stimulate tissue and organ regeneration (Hoang et al., 2022). The common stem cells contain three types: mesenchymal stem cells (MSCs), embryonic stem cells (ESCs), and induced pluripotent stem cells (iPSCs) (Tong et al., 2015). MSCs can be isolated from tissues such as teeth, fat, tonsils, and bone marrow and have the potential to self-renew and differentiate into mesodermal lineages, including adipocytes, muscle, chondrocytes, and osteoblasts, however, clinical therapeutic efficacy is debatable (Hoang et al., 2022). ESCs are pluripotent stem cells produced from the inner cell mass of the blastocyst, but their derivation from human embryos poses various ethical difficulties that limit research into their clinical applications (Clark et al., 2021). iPSCs are stem cells that reprogram adult somatic cells back to their original state; nevertheless, cells produced from iPSC have a risk of causing tumor formation because of reprogramming factors involved with tumor development (Hu et al., 2015).

Cellular scaffolds are artificial temporary platforms used to support, repair, or enhance the structural properties of regenerating tissues (Reddy et al., 2021). To ensure proper tissue function after repair, cellular scaffolds are needed to replace defects or mimic organ or tissue structures in a three-dimensional manner. In general, the selection of a suitable cellular scaffold requires consideration of material properties such as biocompatibility, degradability, mechanical properties, pore size, and osseointegration to ensure therapeutic efficacy (Prasadh and Wong, 2018).

Growth factors are small molecules that transmit signals between cells, such as hormones and mediators (Qu MY. et al., 2020). In tissue regeneration, these growth factors initiate different cellular pathways (Aguilar et al., 2019) by binding to specific receptors and regulate cell proliferation, migration and differentiation by triggering cellular signaling cascades (Lee et al., 2011). However, growth factors have a very short biological half-life and are easily degraded or inactivated (De Witte et al., 2018), so there are many limitations when applying them to tissue engineering. In addition, inappropriate concentrations of growth factors can cause uncontrolled differentiation of stem cells (Zhou et al., 2016) or even carcinogenesis (Tian et al., 2017). Therefore, encapsulation of growth factors in a polymer matrix has become a solution to these problems, a method that both protects growth factors from degradation and regulates the concentration of local growth factor release per unit of time (Qu MY. et al., 2020).

Under this definition, appropriate stem cells and scaffolds are the foundations and starting points for tissue engineering. In recent years, dental pulp stem cells (DPSC) have been intensively investigated as MSC-like cells with multidirectional differentiation potential and strong proliferative capacity for applications in regenerative medicine. DPSC are derived from the neural crest (Dupin et al., 2018; Hall, 2018; Mattei et al., 2021), localized in the dental pulp tissue, and are able to differentiate into a variety of cell types, e.g., endodermal, mesodermal, and ectodermal cells like osteoblasts, adipocytes, chondrocytes, neuronal cells, myoblasts, and islet-like cells (Chang et al., 2014; Kawashima et al., 2017; Kichenbrand et al., 2019; Ji et al., 2020; Longoni et al., 2020). DPSC express typical surface markers in MSCs, including CD 44, CD 73, CD 90 and CD 105 (Delle Monache et al., 2019). Although, there are various limitations to the clinical application of MSCs involving DPSC, such as high cost, problems with cell handling and long-term preservation (Wang et al., 2022). Moreover, it has been suggested by some scientists that implanted MSCs are difficult to survive in the long term (Ogata et al., 2022). Nevertheless, DPSC have the advantages of non-invasiveness, easy isolation and high proliferation rate compared to other sources of stem cells, and therefore remain ideal stem cell candidates in regenerative medicine (Delle Monache et al., 2019). More importantly, to reconstruct tissues and organs, good blood flow reconstruction provided by functional vessels is essential. It has been found that DPSC also have the ability to differentiate into perivascular spectrum cells, including endothelial and pericytes, promoting vascularization (Mattei et al., 2021), which can effectively improve the survival of reconstructed organs. Thus, dental pulp stem cells have a promising future in reconstructive and regenerative medicine and dentistry (Yamada et al., 2019).

The application of cellular scaffolds is important for tissue engineering as they support and promote the growth of regenerating cells (Yi et al., 2017), which can effectively improve the efficiency of tissue engineering. Some scaffold materials not only promote cell proliferation, differentiation and adhesion, but also provide material transport functions and temporary 3D mechanical support, which facilitates new tissue formation around the scaffold (Abbasian et al., 2019). Biomaterials used to fabricate scaffolds must have the right biological and chemical properties to induce molecular recognition of cells and promote cell proliferation and differentiation (Moorthy et al., 2021). However, the choice of biomaterials must be adapted to the purpose of tissue engineering. For different types and structures of regenerated tissues, the physicochemical properties, exposed surface area, pore distribution and porosity of the biomaterials should be taken into account to ensure ideal structural characteristics and suitable formation rate of the nascent tissues (Babaie and Bhaduri, 2018).

Most regenerative treatments and tissue reconstruction involve invasive surgical procedures, and trans- and implantation of biomaterials. Therefore, infections are an immanent surgical risk for these procedures (Perrault et al., 2022). To reduce the incidence of infection, several approaches have been used to control surgical-associated infections, including preoperative prophylactic antibiotics (Salgado-Peralvo et al., 2021), skin disinfection preparation (Sarmey et al., 2015), surgical site antibiotic irrigation (Yao et al., 2018), intraoperative warming (Kumin et al., 2018) and postoperative antibiotics (de Jonge et al., 2020).

Tetracycline hydrochloride (TCH) is an inexpensive antibiotic that was discovered in the late 1940s and exhibits an antimicrobial activity against a wide range of microorganisms including Gram-positive and Gram-negative bacteria, *Chlamydia*, and *Mycoplasma* (Chopra and Roberts, 2001). Several recent studies have combined tetracycline with various biomaterials as scaffolds to develop clinical medical materials that can indirectly promote wound healing by excellent cytocompatibility and anti-inflammatory effects (Shao et al., 2016; Ghorbani et al., 2020). It is worth noting, however, that this good performance is not unique to tetracycline biomaterials. Some studies have also tried to form cellular scaffolds with other kinds of antibiotics such as rifampicin, gentamicin and vancomycin in combination with biomaterials, again obtaining desirable results (Roth et al., 2019; Lee et al., 2020; Padrao et al., 2021). However, in such studies, there is a lack of corresponding data to observe the effect of different concentrations of various types of antibiotics on stem cells. If such new cell scaffolds are to be used for tissue engineering in the future, data on the effects of the corresponding drugs on the metabolism of different types of stem cells are essential, which is a common limitation at this stage of research on such new materials, and a large amount of data in this area will be needed in the future to support their clinical application. These new materials have all shown good antimicrobial activity and biocompatibility including tetracycline. Although the inappropriate use of tetracycline poses some problems (Moullan et al., 2015; Chukwudi, 2016), more experiments have proved that tetracycline is one of the ideal candidates as a cellular scaffold-drug delivery system because of its good biocompatibility and broad-spectrum antimicrobial capacity (Dayaghi et al., 2019). Its promising application in regenerative medicine has been confirmed by several *in vitro* and *in vivo* experimental models (Yao et al., 2014; El-Naggar et al., 2016). In addition, the risk of antibiotic resistance can be substantially reduced by precise application at the target organ site, as the cellular scaffold-drug delivery system allows the application of antibiotics at a reduced dose (Sangtani et al., 2018). Therefore, further studies on the effects of tetracycline on stem cell proliferation and differentiation are necessary in order to facilitate its successful application in tissue engineering in the future. The aim of the present study was to analyze the effects of different concentrations of TCH on DPSC metabolism and thus providing some reference for the application of tetracycline-based drugs and biomaterials in tissue engineering.

## 2 Materials and methods

### 2.1 Collection of samples

Three impacted third molars of healthy teens between the ages of 15 and 19 were acquired in the University Medical Center Hamburg-Eppendorf between September 2021 and October 2021. The medical chamber of Hamburg’s institutional review board approved the experimental protocol (IRB-vote # REC 1712/5/2008). Prior to surgery, patients and their legal guardians signed informed consent papers and were instructed to gargle for 3 minutes with 1% H2O2 solution. Furthermore, during the whole process of operations, the normal sterilizing protocols were followed. The third molars were then extracted and then put into DMEM (Cat. NO. 41965–049, Gibco, Loughborough, UK) at 4°C with 10% FBS (Cat. NO. 10500–064, Gibco, Paisley, UK), penicillin and streptomycin (Cat. NO. 15140–148, Gibco, Paisley, UK).

### 2.2 Cell culture

As described in a previous study, a high-speed turbine was used to split collected tooth and remove dental pulp (Wang et al., 2022). The pulp tissue was then sliced into 0.5–1 mm^3^ tissue pieces using ophthalmic scissors, placed in 24-well cell culture plates and 600 μL DMEM culture medium (containing 10% FBS and 100 U/mL Pen/Strep) was added into each well. The tissue was put into a 5% CO2 incubator at 37°C. The condition of the cells was assessed every day under the microscope. When the colony confluence reached an optimal level (≥80%), cells were isolated by digestion with 0.05% trypsin (Cat. NO. 25300–054, Gibco, Paisley, UK) and passaged (Wang et al., 2022). Dental pulp cells of the 3rd-5th generations were selected for the experiments.

### 2.3 Flow cytometry

3rd generation DPSC were prepared for the experiment of flow cytometry. Specific PE-coupled antibodies were selected, including CD34, CD45, CD73, CD90 and CD105. After adding 2 × 10^5^ cells per tube and centrifuging, of dye mixture which contained live/dead dye and antibodies were added and reacted for 20 min. Then the cells were fully washed with PBS and measured by BD LSRFortessa Cell Analyzer (Becton Dickinson Bioscienc, Becton, United States) and BD FACSDiva software V6.1 (Becton Dickinson Bioscience, United States). FlowJo software V10.0 was then used to analyze the data of flow cytometry (Treestar Inc., OR, United States) (Wang et al., 2022).

### 2.4 Antibiotic preparation

Tetracycline (Sigma-Aldrich, St. Louis, United States) at concentrations of 0, 25, 50, 100, 250, 500 μg/mL, drug-containing culture medium were prepared by dissolving tetracycline powder into DMEM cell culture medium for cell survival rate, colony-forming efficiency, cell migration test and proliferation ability test. Osteogenic induction solution and adipogenic induction solution containing the above concentrations of tetracycline were prepared specifically. The composition of each kind of solutions is shown in Table 1.

**TABLE 1:** Information on the composition of antibiotic solutions used for different experiments.

TCH final concentration	0 μg/mL	25 μg/mL	50 μg/mL	100 μg/mL	250 μg/mL	500 μg/mL
Experimental usage						
**Cell Morphology**	TCH + DMEM +10% FBS +100 U/mL Pen/Strep					
**Cell Survival Rate**	TCH + DMEM +10% FBS +100 U/mL Pen/Strep					
**Cell Proliferation**	TCH + DMEM +10% FBS +100 U/mL Pen/Strep					
**Cell Migration**	TCH + DMEM +100 U/mL Pen/Strep					
**Osteogenic Differentiation**	TCH + DMEM +10% FBS +100 U/mL Pen/Strep +10 mmol/L glycerophosphate +50 μmol/L ascorbic acid +0.1 μmol/L dexamethasone					
**Adipogenic Differentiation**	TCH + DMEM +10% FBS+ 100 U/mL Pen/Strep +10 μg/mL insulin +0.5 mmol/L IBMX +200 μmol/L indomethacin +1 μmol/L dexamethasone					

That the bold values are a list of the final concentrations of TCH added to the experiment. In addition to the base culture solution used for each experiment mentioned in the table, we added different amounts of TCH drug to achieve the corresponding final concentration of culture solution used for each experiment.

### 2.5 Assessment of cellular morphology

DPSC were cultured in 12-well plates (4 × 10^5^ cells/well). After the DPSC were completely adhered to the walls, medium containing (0–500 μg/mL) of TCH was added and cultured for 24 h. DPSC were then fixed by 4% formaldehyde solution, soaked in 0.5% Triton-X-100, then stained with 1:1,000 Phalloidin (Cat. NO. A12379, Thermo Fisher Scientific, United States) solution for 1 h. The morphology of each group of cells was observed by two trained and calibrated experimenters by a fluorescence microscope (Nikon ECLIPSE Ti-S/L100, Germany).

### 2.6 Cell survival rate

The 3rd generation DPSC cultured in each concentration of tetracycline were seeded at a density of 4 × 10^4^/mL on TCC (tissue culture coverslips, Sarstedt, Nümbrecht, Germany), and incubated for 8 h in the incubator. For live-dead staining, propidium iodide at 50 μg/mL and fluorescein diacetate at 20 μg/mL were used for each sample. After 3-min staining, the samples were rinsed with DPBS and examined under the same fluorescence microscope mentioned before. After taking 3 photographs of each concentration of DPSC, the photographs were analyzed using Image J software, and the number of live cells (green-stained), the number of dead cells (red-stained), and the total number of cells (green-stained + red-stained) under the microscope were calculated and counted in each photograph. After that, the cell survival rate of each concentration group was calculated separately with the following equation:

Cell survival rate = number of green-stained cells/(number of green-stained cells + number of red-stained cells) × 100%

### 2.7 Proliferation testing with MTS assay

4th generation DPSC were plated in 96-well plates (1 × 10^4^/mL, 100ul/well), and incubated at 37°C. Cell proliferation was detected every day using MTS assay kit (Cat. NO. G1111, Promega, United States) for a total of 8 consecutive days, with replacement of the DMEM culture medium containing tetracycline 24 h after plating. Cells in 3 wells of each sample for each concentration group were set for the test. 20 μL of MTS mixture was used for each well. 3 h later, the absorbance value of each group was measured by a microplate reader.

### 2.8 Cell migration rate

Fourth-generation DPSC were plated at a density of 7.5× 10^4^/mL, corresponding to 1.5× 10^5^ cells per well. After 24-h incubation, center of each well was lined with a 20ul pipette tip and rinse three times with PBS to remove residual suspended cells. Afterwards, serum-free DMEM medium containing different concentrations of TCH was added. Photographs were taken at 0h, 12h and 24 h respectively, and the blank areas were calculated using ImageJ software, and the data were normalized to calculate the cell migration rate.

### 2.9 Assessment of differentiation potential

#### 2.9.1 Osteogenic differentiation

Fifth-generation DPSC were inoculated in 6-well plates at 4 × 10^4^ per well for culture. The concentration of each group of cell suspensions was calculated three times with an EVE automated cell counter before culturing, and after normalizing the concentration of each group of cells, the cell suspensions were added into 6-well plates in the same volume, ensuring that the same number of cells and volume of culture medium were added to each well. Cell growth was observed under a microscope daily, and three randomly selected positions of each group of cells were photographed, and the average confluency of each group of cells was calculated respectively using ImageJ software. After cells reached 60%–70% confluency, they were grown in osteogenic induction medium containing various concentrations of tetracycline for 3 weeks, with fresh induction medium changes every 3 days and osteogenic differentiation was conducted in triplicates. 3 weeks later, DPSC were fixed with paraformaldehyde and dyed using 0.1% Alizarin Red S before observation.

#### 2.9.2 Quantitative analysis of osteogenic differentiation

The dyed DPSC were rinsed with DPBS. Thereafter, 750 μL of acetic acid (10%) was added to completely dissolve the red-dyed calcareous nodules. Then750 μl of ammonium hydroxide (10%) was added. Supernatant was added in a 96-well plate and detected at 405 nm by a microplate reader. Each sample was set up in triplicate at the time of testing.

#### 2.9.3 Adipogenic differentiation

Fifth-generation DPSC were inoculated in 6-well plates at 4 × 10^4^ per well for culture. The concentration of each group of cell suspensions was calculated three times with an EVE automated cell counter (NanoEntek, Seoul, Korea) before culturing, and after normalizing the concentration of each group of cells, the cell suspensions were added into 6-well plates in the same volume, ensuring that the same number of cells and volume of culture medium were added to each well. Cell growth was observed under a microscope daily, and three randomly selected positions of each group of cells were photographed, and the average confluency of each group of cells was calculated respectively using ImageJ software. After cells reached 60%–70% confluency, the medium was replaced by adipogenic differentiation medium with various concentrations of tetracycline, with fresh induction medium changes every 3 days and adipogenic differentiation was conducted in triplicates. 3 weeks later, DPSC were fixed with paraformaldehyde and dyed using 0.5% Oil Red O solution before observation.

#### 2.9.4 Quantitative analysis of adipogenic differentiation

The dyed DPSC were rinsed with DPBS. 1 mL isopropanol was added to dissolve the lipid droplets until the solution was uniformly colored. The supernatant was then added in a 96-well plate and detected at 540 nm by a microplate reader. Each sample was set up in triplicate at the time of testing.

### 2.10 Gene expression

RNA was extracted with TRIzol reagent (Cat. NO. 15596026, Ambion, TX, Austin, United States). Then it was reverse transcribed using GoScriptTM RT kit. PCR was performed with the Luna^®^ universal One-Step RT-qPCR kit. Lipoprotein Lipase (LPL), Peroxisome proliferator-activated receptor-γ (PPAR-γ), alkaline phosphatase (ALP), runt-related transcription factor 2 (RUNX2), type I collagen (COL I) and osteocalcin (OCN) were chosen as specific differentiation genes. GAPDH was chosen as reference housekeeping gene. Cq results was computed by 2^−ΔΔCt^ after normalized (Alaidaroos et al., 2021). The primer sequences was shown as Table 2 below.

**TABLE 2:** Primer sequences of adipogenic and osteogenic-induced gene expression.

Primer	Direction	Sequence	Length of products (bp)
LPL	Forward	ACA​AGA​GAG​AAC​CAG​ACT​CCA​A	76
	Reverse	GCG​GAC​ACT​GGG​TAA​TGC​T	
PPAR-γ	Forward	GGG​ATC​AGC​TCC​GTG​GAT​CT	186
	Reverse	TGC​ACT​TTG​GTA​CTC​TTG​AAG​TT	
ALP	Forward	ACT​GGT​ACT​CAG​ACA​ACG​AGA​T	97
	Reverse	ACG​TCA​ATG​TCC​CTG​ATG​TTA​TG	
RUNX 2	Forward	TGG​TTA​CTG​TCA​TGG​CGG​GTA	97
	Reverse	TCT​CAG​ATC​GTT​GAA​CCT​TGC​TA	
Type I collagen	Forward	GGA​CAC​AAT​GGA​TTG​CAA​GG	441
	Reverse	AAC​CAC​TGC​TCC​ACT​CTG​G	
Osteocalcin	Forward	GGC​GCT​ACC​TGT​ATC​AAT​GG	110
	Reverse	GTG​GTC​AGC​CAA​CTC​GTC​A	
GAPDH	Forward	GAG​TCA​ACG​GAT​TTG​GTC​GT	185
	Reverse	GAC​AAG​CTT​CCC​GTT​CTC​AG	

### 2.11 Statistical analysis

An analysis of the variation in mean values within each group was done using a Student’s t-test. A *p*-value of less than 0.05 was used. SPSS 25.0 (SPSS Inc., IL, Chicago, United States) and GraphPad Prism 9.0.0 (GraphPad Software, CA, San Diego, United States) were used to analyze the data.

## 3 Results

### 3.1 Identification of specific stem cell markers on DPSC at 3^rd^ generation

**FIGURE 1:**
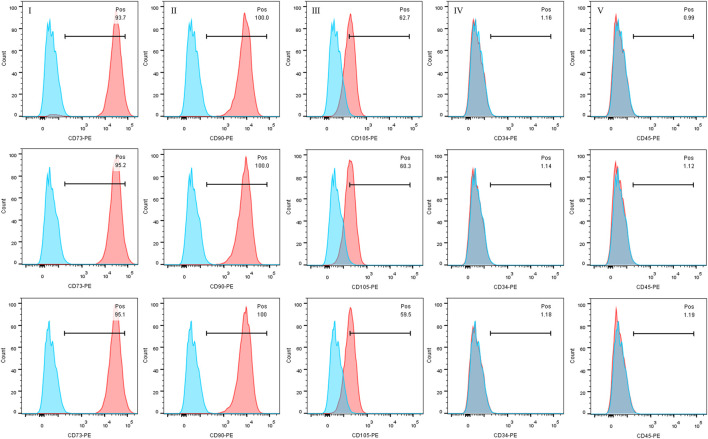
Identification of MSCs by flow cytometry. The expression of CD73, CD90 and CD105 was positive, otherwise CD45 and CD34 expression was negative.

CD34, CD45, CD73, CD90 and CD105 were selected and detected as specific markers before experiments. The surface antigen expression of the three groups of samples were: CD34: (94.67 ± 0.8386) %, CD45: (100.0 ± 0.000) %, CD73: (60.83 ± 1.665) %, CD90: (1.160 ± 0.02000) % and CD105: (1.100 ± 0.1015) %. CD73, CD90 and CD105 were abundantly expressed, while CD34 and CD45 were not. (Figure 1).

### 3.2 The impact of different TCH concentrations on the morphology of DPSC at 3^rd^ generation

**FIGURE 2:**
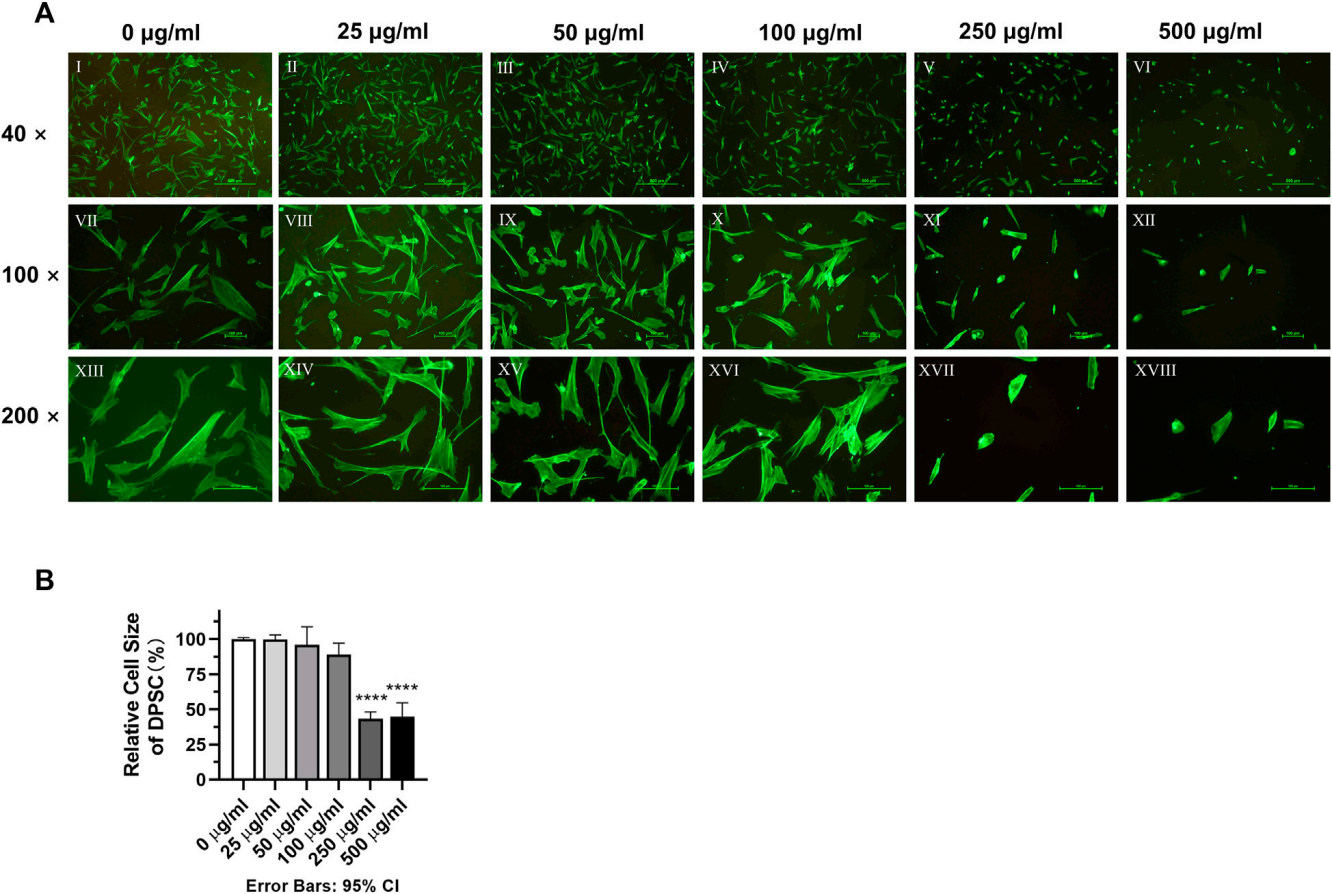
Morphology of dental pulp cells in different concentration TCH groups. **(A)** Ⅰ–Ⅵ: fluorescence microscope (×40). Ⅶ–Ⅻ: fluorescence microscope (×100). XIII–XVIII: fluorescence microscope (×200). **(B)** Relative size statistics of dental pulp cells in different concentrations of TCH solutions (n = 3). The one-way ANOVA was used in multiple group data analysis. *∗∗∗∗ p < 0.0001* vs. *control* (*0* μg/mL)*.*

DPSC were cultured in different concentrations of TCH culture medium for 24 h. Then they were stained with Phalloidin, photographed and observed for each group of cell morphology. After assessment by two trained examiners (W.W and J.S), the Cohen’s kappa coefficients of the 250 μg/mL and 500 μg/mL groups were *κ* = 15.4% and 11.1% respectively, compared to the 0 μg/mL blank group. Whereas the Cohen’s kappa coefficients of the other groups were greater than 80%, indicating that the morphology of DPSC in the 250 μg/mL and 500 μg/mL groups was significantly altered. Phalloidin fluorescence staining results showed that the cells in the 0–100 μg/mL group had a spreading morphology with a triangular or spindle shape. In contrast, the cells of DPSC in the 250–500 μg/mL groups were crinkled and the cell pseudopods were not obvious. After calculation of the cell size in other concentration of TCH conditions separately using the 0 μg/mL concentration group as the control group, it is found that the cells in the 250 μg/mL group and the 500 μg/mL group were significantly reduced. The size of cells in the 250 μg/mL group and 500 μg/mL group was reduced to (43.25 ± 5.055) % and (44.92 ± 9.820) % of the control group, respectively, and the difference was statistically significant (Figure 2B) (*p* < 0.0001).

### 3.3 Effects of different TCH concentrations on cell survival rates of DPSC at 3^rd^ generation after 24-h incubation

**FIGURE 3:**
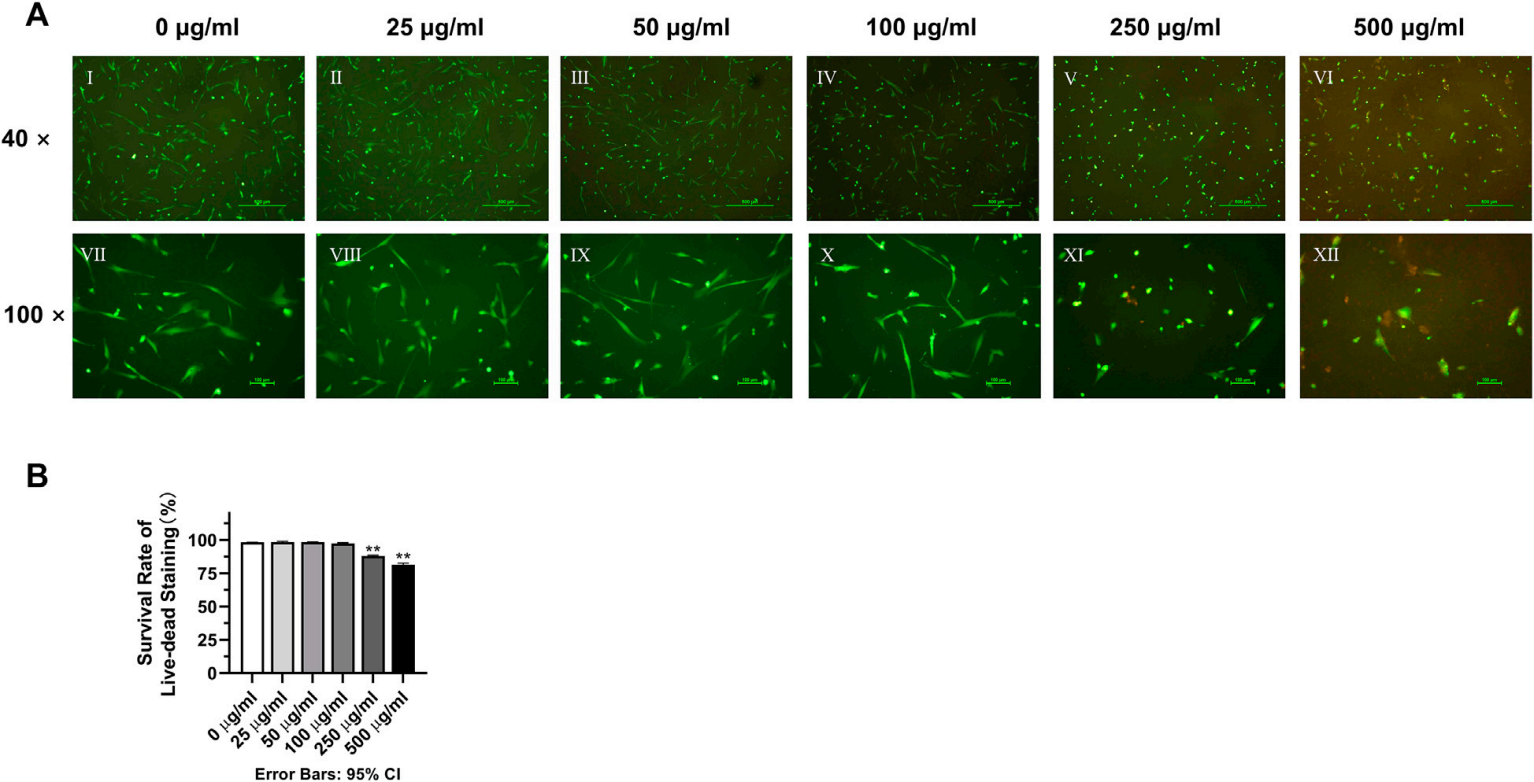
Effects of various concentrations of TCH on the cell survival rate. **(A)** Ⅰ–Ⅵ: 0–500 μg/mL (×40). Ⅶ–Ⅻ: 0–500 μg/mL (×100); **(B)** The survival rate of live–dead staining in each group (n = 3). The one-way ANOVA was used in multiple group data analysis. **p < 0.01 vs. *control* (*0* μg/mL)*.*

It can be clearly seen from the live-dead cell staining results that a higher number of PI red-stained dead cells appeared in the 250 μg/mL and 500 μg/mL groups (Figure 3A). The results of counting and statistical analysis showed that the percentage of live cells in the 250 μg/mL (88.01% ± 0.5834%) and 500 μg/mL (81.32% ± 1.285%) groups was lower than that in the 0 μg/mL blank control group (98.15% ± 0.1805%), and the difference was statistically significant (Figure 3B) (*p < 0.01*).

### 3.4 Dose dependent effects of TCH on primary cell proliferation of DPSC at 4^th^ generation

**FIGURE 4:**
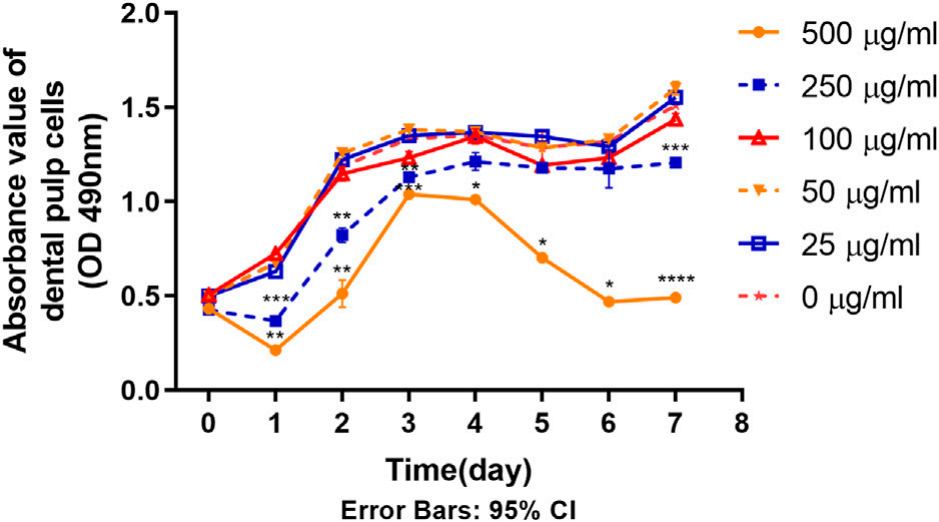
Proliferation ability of primary dental pulp cells in each group (n = 3). The two-way ANOVA was used in multiple group data analysis. *∗ p < 0.05, ∗∗ p < 0.01, ∗∗∗ p < 0.001, ∗∗∗∗ p < 0.0001* vs. *control* (*0* μg/mL)*.*

As shown in Figure 4, the proliferation ability of DPSC in the 250 μg/mL and 500 μg/mL groups was significantly inhibited after the contact with TCH on day 1. From the 7 -day proliferation curve, generally, the rising trend of cell proliferation curves in 250 μg/mL and 500 μg/mL groups was lower than that of the blank control group (0 μg/mL) in some timepoints. On day 1–3 and day 7, the absorbance value in 250 μg/mL was significantly lower than that in 0 μg/mL group. On day 1–7, the absorbance value in 500 μg/mL was significantly lower than that in 0 μg/mL group. The difference is statistically significant (*p < 0.05*). While the proliferation capacity of DPSC in the remaining concentration groups was unaffected.

### 3.5 Dose dependent effects of TCH on the primary cell migration ability of DPSC at 4^th^ generation after 24-h incubation

**FIGURE 5:**
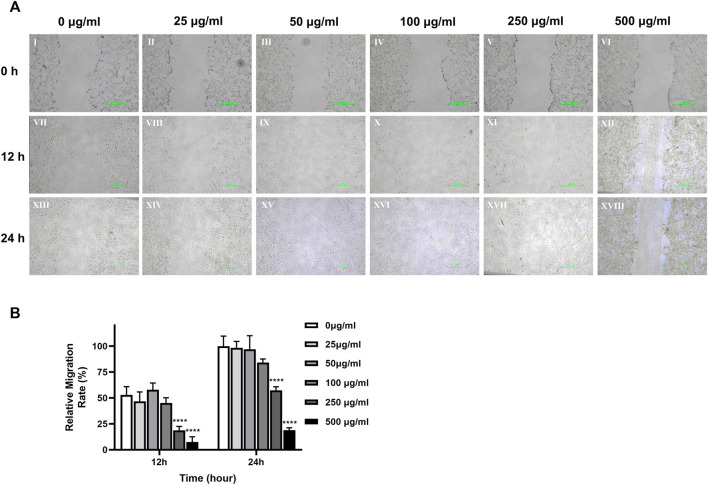
Cell migration ability of dental pulp cells in different concentration TCH groups. **(A)** Photographs of monolayer DPSC were taken at 0h, 12h and 24 h after scratching and adding different concentrations of TCH. Ⅰ–Ⅵ: 0–500 μg/mL (×40). Ⅶ–Ⅻ: 0–500 μg/mL (×100). **(B)** The area of the scratches in different groups at each time point was measured using ImageJ software, which was normalized to calculate the cell migration rate of each group relative to 0 h (n = 3). The two-way ANOVA was used in multiple group data analysis. *∗∗∗∗ p < 0.0001* vs. *control* (*0* μg/mL).

The photographs in Figure 5A show that the cell movement was relatively slow in the 500 μg/mL and 250 μg/mL concentration groups. Normalized calculations of the dimension of the scratch experiments showed that 500 μg/mL and 250 μg/mL concentrations of TCH significantly decelerated the cell migration rate compared to the 0 μg/mL concentration blank control group. At 12 h, the cell migration rate was 7.57% ± 4.05% and 18.60% ± 3.20% for the 500 μg/mL and 250 μg/mL concentration groups respectively, which was significantly lower than that of the 0 μg/mL concentration group (52.91% ± 6.55%). At 24h, the cell migration rate was 18.99% ± 1.84% and 57.41% ± 2.78% in the 500 μg/mL and 250 μg/mL concentration groups respectively, which was still significantly lower than that in the 0 μg/mL concentration group (100.00% ± 7.97%). The experimental results were statistically different (*p < 0.05*).

### 3.6 Effects of TCH on the osteogenic differentiation potential of DPSC at 5^th^ generation after 3-week induction

**FIGURE 6:**
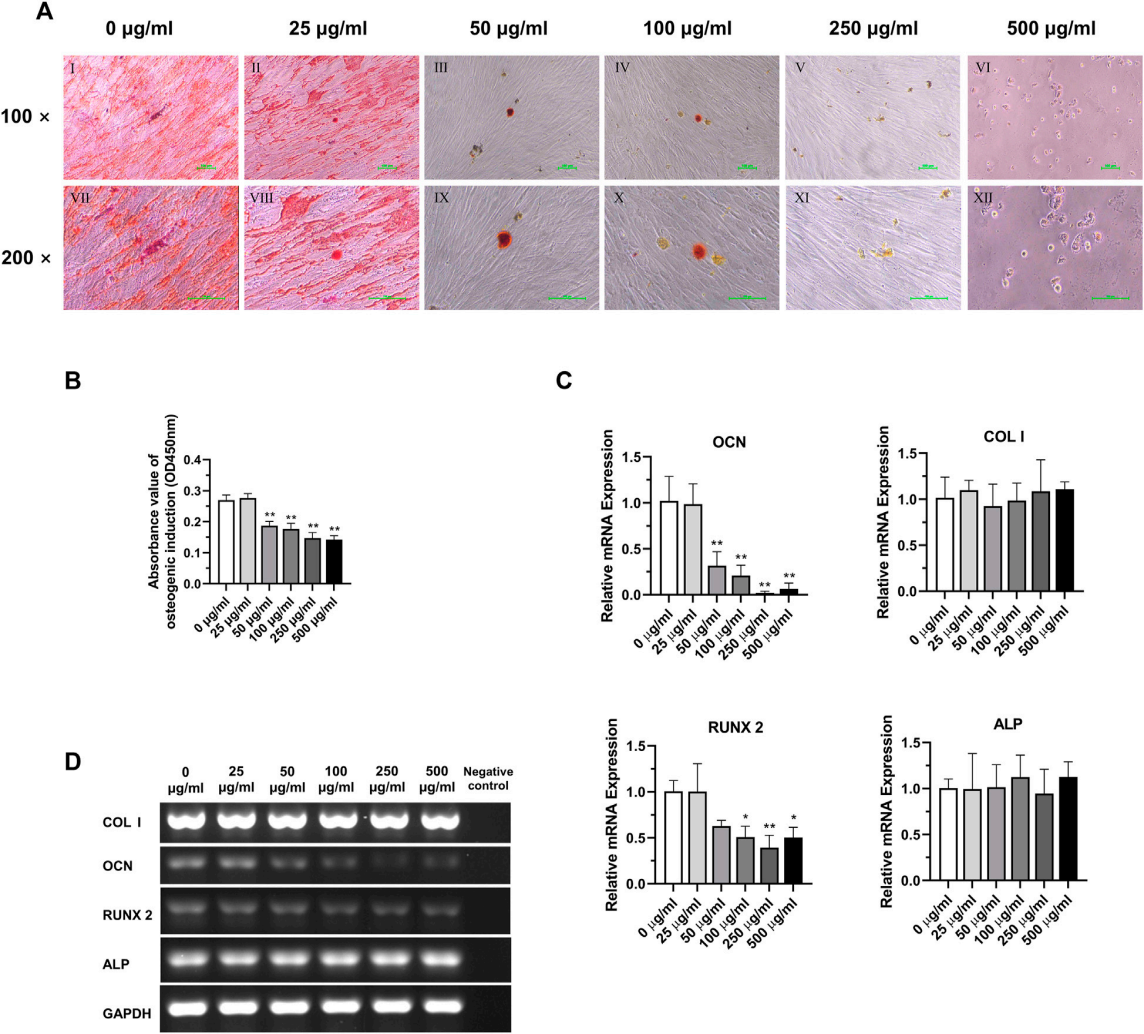
Osteogenic differentiation potential of DPSC in TCH. **(A)** Alizarin Red staining after osteogenic differentiation results of DPSC. Ⅰ–Ⅵ: 0–500 μg/mL (×100). Ⅶ–Ⅻ: 0–500 μg/mL (×200). **(B)** The absorbance value at 450 nm of dissolved solution after Alizarin Red S staining in different groups (n = 3). **(C)** The relative osteogenic mRNA expression value in different groups (n = 3). **(D)** Gel electrophoresis results of osteogenic gene expression in different groups, GAPDH was selected as reference gene. The one-way ANOVA was used in multiple group data analysis. *∗ p < 0.05, ∗∗ p < 0.01* vs. *control* (*0* μg/mL).

The results of osteogenic induction showed a significant inhibition of osteogenic differentiation function of DPSC in the concentration of TCH solution above 50 μg/mL 0 μg/mL and 25 μg/mL concentration groups showed significant red staining of bone nodules. However, in the concentration group above 50 μg/mL, the number of calcium nodule was significantly reduced, and the staining was not obvious due to mineralization incompleteness. 500 μg/mL concentration group of DPSC even showed a large reduction of cells (Figure 6A). The absorbance of the calcium nodule lysate decreased significantly in the group with concentrations above 50 μg/mL, and the difference was statistically significant (Figure 6B) (*p* < 0.01). The results of the detection of the main osteogenic genes in each group showed that the expression of OCN was significantly reduced in the group with the concentration above 50 μg/mL, and the expression of Runx2 was significantly reduced in the group with the concentration above 100 μg/mL, and the difference was statistically significant (Figures 6C,D) (*p < 0.05*)*,* while the expression of ALP and COL Ⅰ had no statistical difference among those groups (Figures 6C,D) (*p > 0.05*).

### 3.7 Effects of TCH on the adipogenic differentiation potential of DPSC at 5^th^ generation after 3-week induction

**FIGURE 7:**
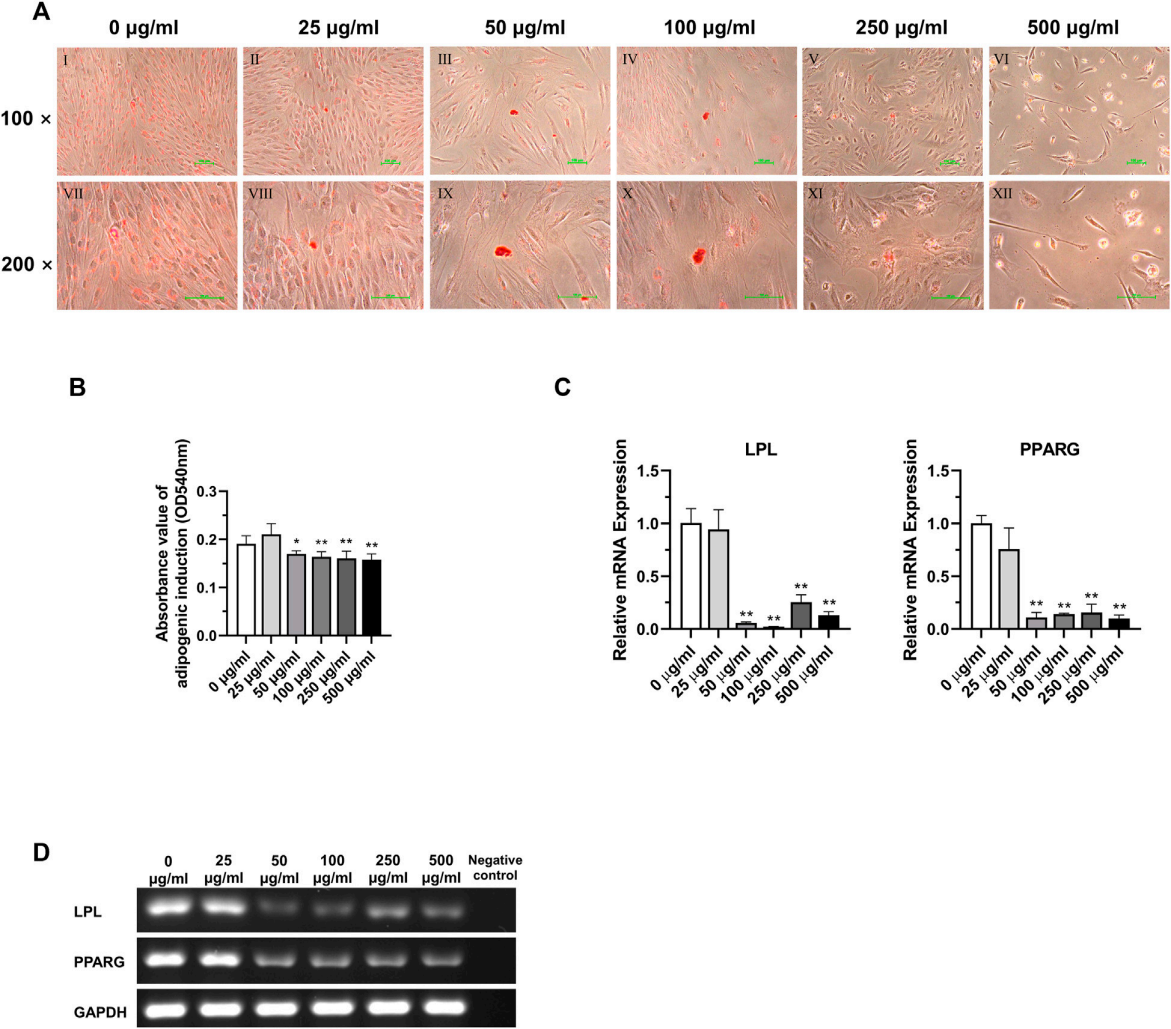
Adipogenic differentiation potential of DPSC in TCH. **(A)** Oil Red O staining after adipogenic differentiation results of DPSC. Ⅰ–Ⅵ: 0–500 μg/mL (×100). Ⅶ–Ⅻ: 0–500 μg/mL (×200). **(B)** The absorbance value at 540 nm of dissolved solution after Oil Red O staining in different groups (n = 3). **(C)** The relative osteogenic mRNA expression value in different groups (n = 3). **(D)** Gel electrophoresis results of adipogenic gene expression in different groups, GAPDH was selected as reference gene. The one-way ANOVA was used in multiple group data analysis. ∗ *p < 0.05,* ∗∗ *p < 0.01* vs. *control* (*0* μg/mL)*.*

According to the analysis of the results of adipogenic induction, tetracycline induction solution at concentrations above 50 μg/mL had an inhibitory effect on the adipogenic differentiation ability of DPSC. Lipid droplets around the nucleus were more obvious in the 0 μg/mL and 25 μg/mL concentration groups. In the concentration groups above 50 μg/mL, the intracellular lipid droplets were significantly reduced. In addition, simultaneous application of lipogenic induction solution and TCH at concentrations above 50 μg/mL resulted in altered cell morphology and a significant decrease in the number of cells in DPSC (Figure 7A). Dissolved solution results showed a statistically significant decrease in the absorbance of lipid droplet lysate in the group with concentrations above 50 μg/mL (Figure 7B) (*p* < 0.05). The results of detecting the main lipogenic genes in each group demonstrated that the expression of LPL and PPARG was significantly reduced in the group with concentrations of tetracycline above 50 μg/mL, and the difference was statistically significant (Figures 7C,D) (*p < 0.01*).

## 4 Discussion

The present study analyzed the effects of TCH on dental pulp stem cells (DPSC). To assess the effect of different tetracycline concentrations on the metabolism of DPSC, we collected and isolated DPSC from three-third molars from three donors. First, according to the International Society for Cell Therapy’s (ISCT) guidelines (Viswanathan et al., 2019), the surface antigens of the extracted DPSC were examined and the expression of CD34 and CD45 was found negative, while the expression of CD73, CD90 and CD105 was positive, confirming that the isolated DPSC are MSC-like cells with typical MSC characteristics and stemness (Qu C. et al., 2020). Further experiments including Phalloidin staining, live-dead staining, cell proliferation assay and cell scratching assay of DPSC in different concentrations of TCH demonstrated that TCH at 250 μg/mL and 500 μg/mL groups altered cell morphology and caused cell consolidation, reducing cell viability, proliferation and migration ability. Furthermore, after 21 days of induction, we noted a significant inhibition of osteogenic and adipogenic differentiation of DPSC above 50 μg/mL of tetracycline environment. This was strongly supported by the marked reduction in the expression of two key osteogenic genes, namely, OCN and RUNX2 and adipogenic genes involving LPL and PPARG. Therefore, it is demonstrated that tetracycline at a concentration of 50 μg/mL or more effectively reduced the differentiation ability of DPSC in the osteogenic and lipogenic directions.

There are three important components of tissue engineering: finding a suitable source of stem cells, developing a biocompatible cellular scaffold and using the corresponding cytokines to induce the occurrence of signals according to the therapeutic purpose. Among them, stem cells and scaffolds are the basis of tissue engineering. Human adult-derived mesenchymal stem cells (MSCs) are considered an ideal source for regenerative medicine and tissue engineering due to their self-renewal and multidirectional differentiation potential, and because they present few of the ethical concerns associated with embryonic stem cells (Anitua et al., 2018). Common sources of MSC tissue include adipose tissue (Ong et al., 2021), bone marrow (Karaoz et al., 2011), umbilical cord (Lyons and Mattei, 2019), placenta (Shin et al., 2021), *etc.* Unfortunately, although MSCs from the above sources have good tissue engineering properties, collecting them often requires highly invasive manipulation, leading to a range of complications at the donor site (Ducret et al., 2015; Kang et al., 2016). In comparison, pulp-derived mesenchymal stem cells have more advantages, such as they are easier to obtain, less costly and less damaging to the donor site, as they are usually derived from third molars (Ledesma-Martinez et al., 2016), multiple teeth (Ferrua et al., 2017) or teeth that need to be extracted for orthodontic purposes (Al Madhoun et al., 2021). Existing studies on DPSC are relatively well established, and it is known that DPSC have high proliferation rates and low immunogenicity, and have the potential for multidirectional differentiation, including differentiation into osteoblasts, adipocytes, chondrocytes, neuronal cells (Goorha et al., 2022), and hepatocytes (Ishkitiev et al., 2010; Ishkitiev et al., 2013; Song et al., 2016; Yoshida et al., 2016; Zhang et al., 2016). A study showed that DPSC have a greater proliferative capacity compared to human bone marrow mesenchymal stem cells (BMMSCs) (Alge et al., 2010). Another study corroborated the idea that the expression level of telomerase in human DPSC is higher than that in BMMSCs, which allows telomeres in DPSC to maintain higher activity, resulting in a greater proliferation and colony formation capacity of DPSC (Sonoyama et al., 2006). Therefore, these excellent biological properties of DPSC determine them to be excellent candidates for tissue engineering. In fact, several animal experiments using DPSC to repair functional defects in tissues have demonstrated the promising application of DPSC in tissue engineering. Dong et al. injected DPSC *in situ* into a rat model of intervertebral disc (IVD) degeneration and found that they protected the structural integrity of the rat IVD and reduced extracellular matrix lectures (Dong et al., 2022). Inada et al. transplanted pancreatic β-like cells obtained after induction of DPSC into a rat model of diabetes mellitus and obtained good therapeutic results (Inada et al., 2022). Gonzaga et al. used DPSC for intraperitoneal treatment of aplastic anemia model mice and found that DPSC provided rapid support for hematopoiesis and hematopoiesis recovered after 6 months (Gonzaga et al., 2022). Wenceslau et al. used DPSC intravenously to treat rats with Huntington’s chorea model, resulting in a significant increase in the secretion of brain-derived neurotrophic factor, which effectively promoted neuroprotection and neurogenesis (Wenceslau et al., 2022). There are other experimental studies in which DPSC were injected intravenously into animal models for the treatment of diseases such as ischemic stroke (Zhang et al., 2018), retinal degeneration (Lam et al., 2021), diabetic neuropathy (Datta et al., 2017) and Sjögren’s syndrome (Matsumura-Kawashima et al., 2021), and ideal results were obtained. However, as mentioned before, tissue engineering mostly involves invasive procedures and requires antibiotics to avoid opportunistic infections. In addition, intravenous injection of DPSC involves the migration and homing ability of the cells. If inappropriate concentrations of TCH are applied to avoid infection, this may prevent DPSC from reaching the target organ, which may adversely affect the clinical outcome. Therefore, the choice of antibiotic type and concentration in stem cell injection therapy should also be considered as one of the factors for clinical efficacy. Based on the data from the present study, when TCH is combined with DPSC intravenously for tissue engineering, the local drug concentration in the target organ should be lower than 100 μg/mL so that the migration and homing ability of DPSC will not be affected, thus avoiding adverse effects on the clinical outcome.

The use of antibiotics in tissue engineering is almost inevitable in order to reduce postoperative infections resulting from invasive manipulation and implantation of biomaterials. However, the increase in pathogen resistance to early-detected antimicrobials is posing a major threat to human health (Livermore, 2009). On the other hand, studies have found that the availability of newer antibiotics is beginning to decline (Boucher et al., 2009; Theuretzbacher, 2012). The negative impact of these conditions on clinical outcomes is undeniable. Therefore, there is a need for effective antibiotic stewardship and optimization of the use of antibiotics used intraoperatively. World Health Organization (WHO) has proposed a “One Health” global program to address antimicrobial resistance. This includes increasing awareness and understanding of antimicrobial resistance, increasing investment in new drugs, diagnostic tools, vaccines, and optimizing the use of antimicrobials (Collignon and McEwen, 2019). One effective measure is to combine antibiotics with biomaterials, which can reduce the amount of antibiotics used while ensuring local drug concentrations and reducing the environmental impact of unmetabolized excreted antibiotics. Local antibiotic delivery routes based, for example, on cellular scaffolds have many advantages: the drug can reach higher concentrations at the surgical site while keeping the systemic antibiotic concentrations to a minimum (Dorati et al., 2017a), so most of the adverse effects caused by systemic high-dose antibiotic application, such as hepatotoxicity and nephrotoxicity, can be avoided. In addition, the potential for antibiotic resistance is correspondingly reduced due to the reduced drug dosage. For example, Dorati et al. combined gentamicin with a thermosetting composite stent (ITCS) to create a scaffold system, which effectively reduced the dose of gentamicin used in the treatment of chronic osteomyelitis and was able to reduce the damage to the kidney from gentamicin (Dorati et al., 2017b; De Trizio et al., 2017). Tao et al. combined a chitosan-based thermosensitive hydrogel with vancomycin to create a drug-release scaffold for the treatment of rabbits with osteomyelitis and found a substantial reduction in vancomycin dosage while maintaining the anti-infective effect of the material, resulting in reduced vancomycin ototoxicity and nephrotoxicity (Tao et al., 2020).

TCH is one of the most widely used antibiotics in the treatment of bacterial infections including acnes, periodontal and urinary. It is used in the treatment of various bacterial infections and in the prevention of postoperative infections because of its relatively broad antibacterial spectrum, its activity against a wide range of Gram-positive and Gram-negative bacteria, and its low minimum inhibitory concentration (Chopra and Roberts, 2001), which are often required for tissue engineering applications involving invasive manipulation. Regular systemic administration ensures antimicrobial action through the blood circulation of the drug (Wang et al., 2012; Cibor et al., 2017). However, this route has obvious disadvantages, including toxic effects on other organs and insufficient drug concentrations in the target tissue sites. To address this problem, several researchers in recent years have attempted to combine TCH with various organic polymers, especially degradable ones, to simultaneously function as a cellular scaffold and a drug delivery system. Allafchian et al. combined TCH with polysaccharide aloe vera gel and polyvinyl alcohol to form a cellular scaffold and found good antibacterial activity against *Staphylococcus aureus* and *Bacillus* cereus without affecting cell adhesion and proliferation (Allafchian et al., 2022). Ferreira et al. used a cellular scaffold combining TCH, metronidazole, and electrostatic spinning for the treatment of rats in a periodontitis model and found increased new bone formation, decreased bone loss, and reduced inflammatory response (Ferreira et al., 2021). Ding et al. used a combination of tetracycline, matrix metalloproteinase 2, and strontium-doped calcium polyphosphate scaffold to treat rabbit cranial defects and found good antibacterial and bone regeneration-promoting effects (Ding et al., 2019). Dayaghi et al. used porous magnesium alloy combined with tetracycline as a cellular scaffold and found not only good antimicrobial activity and biocompatibility, but also promoted the degree of differentiation of osteoblasts (Dayaghi et al., 2019). In conclusion, various cellular scaffold materials loaded with tetracycline seem to achieve good results and possess a wide range of application prospects.

While both DPSC and tetracycline-containing cell scaffolds are ideal for tissue engineering, it is noteworthy that unsuitable concentrations of drugs tend to be cytotoxic, thereby compromising the effectiveness of tissue regeneration and differentiation. According to the survey from World Organization for Animal Health (WOAH), the main antimicrobials commonly used worldwide include tetracyclines (37.1%), peptides (15.7%), penicillins (9.8%), macrolides (8.9%), and aminoglycosides (7.8%) (Gochez et al., 2019). TCH is used as a common treatment medicine in many countries (Qodrati et al., 2022), which can inhibit bacterial protein synthesis by binding to the ribosome and interacting with the 16S ribosomal RNA in the 30S ribosomal subunit (Chukwudi, 2016). Nevertheless, antimicrobial resistance is a global public health problem (Shankar and Balasubramanium, 2014). In some regions, the rise of bacterial resistance is very common, and certain bacteria are resistant to almost all types of antibiotics (Laxminarayan et al., 2013; Solomon and Oliver, 2014). Therefore, international agencies, involving World Health Organization (WHO), have developed a series of antibiotic stewardship measures to reduce the risk of microbial resistance (Mendelson and Matsoso, 2015; Public Health Agency of Canada et al., 2015), including reducing the occurrence of infections through effective environmental health and infection prevention measures, and optimizing the use of antimicrobial drugs in human and animal health (Pierce et al., 2020).

To avoid these adverse effects, our experiments investigated the effect of different concentrations of TCH on the cellular metabolism of DPSC and demonstrated that TCH at concentrations higher than 250 μg/mL may not be suitable for application in dental pulp stem cell therapy. The concentration of tetracycline should be further reduced to less than 50 μg/mL when the therapeutic purpose involves the application of differentiation function on DPSC. Relatively speaking, local TCH concentrations at or below 25 μg/mL are safe and do not affect the cellular metabolism of DPSC. However, this should only be used as a reference for the cellular scaffold-drug delivery system or local TCH concentration selection, with the aim of avoiding systemic drug overdose and the effect of other organ metabolism on local drug concentration.

Through this study, our experimental results can provide some reference for the application of tetracycline in tissue engineering to minimize the adverse effects of tetracycline on the growth and differentiation of DPSC. But due to the difference between *in vitro* and *in vivo* microenvironment, it is necessary to apply animal models for the validation of local application of TCH to provide more evidence for the clinical application of the new material. However, because of the limited experimental conditions, we were unable to perform further validation of the experimental findings on animal models. If we could perform animal experiments, we could observe the combined application of both DPSC and TCH according to the proliferation and differentiation requirements of stem cells in different diseases, making the study closer to clinical applications and the experimental results more convincing. Our future studies may continue this topic to deepen the understanding of the effect of tetracycline on the metabolism of DPSC.

## 5 Conclusion

The purpose of our experiments was to investigate the effects of different concentrations of TCH on the growth and metabolism of DPSC. Our experimental results reveal that high concentrations of TCH are detrimental to the metabolism of DPSC. When grown in an environment of TCH above 250 μg/mL, DPSC showed abnormal cell morphology, a decrease in the proportion of viable cells, and a significant decrease in cell proliferation capacity and cell migration rate. When grown in TCH environment above 50 μg/mL, the osteogenic and lipogenic differentiation ability of DPSC was inhibited, and the related osteogenic and lipogenic genes were significantly downregulated. Local TCH concentrations at or below 25 μg/mL are safe and do not affect the cellular metabolism of DPSC, which can be used as a reference for cellular scaffold-drug delivery systems or for local TCH concentration selection. Our experimental findings provide some reference for the use of TCH in tissue engineering: when systemic dosing or the development of new TCH-containing antimicrobial cell scaffold materials is required, it should be noted that TCH at concentrations beyond those described above could potentially cause cytotoxic effects for DPSC and thus affect clinical efficacy. Our study demonstrates a relatively precise range of TCH applications, especially when it involves DPSC in stem cell therapy, and can provide some theoretical basis for tissue engineering.

## Data Availability

The raw data supporting the conclusion of this article will be made available by the authors, without undue reservation.
